# Five novel TTR variants: associated phenotypes and structural consequences

**DOI:** 10.1186/1750-1172-10-S1-P28

**Published:** 2015-11-02

**Authors:** Dorota Rowczenio, Helen Lachmann, Ashutosh Wechalekar, Janet Gilbertson, Marianna Fontana, Carol Whelan, Julian Gillmore, Philip Hawkins

**Affiliations:** 1 National Amyloidosis Centre, University College London, NW3 2PF, London, UK

## Objective

Transthyretin (ATTR) amyloidosis is the commonest form of hereditary amyloidosis. More than 120 amyloidogenic TTR mutations have been described and with few exceptions, these are rare, reported only in isolated kindreds. We describe here the clinical characteristics and disease course in 11 patients with novel TTR mutations. The structural consequence of replacing the wild-type amino acid with variant was examined in silico using PyMOL, an online molecular visualization tool.

## Methods

11 individuals with features raising the possibility of ATTR amyloidosis were referred to the UK NAC for clinical and laboratory investigation.

## Results

Median (range) age at onset of symptoms was 58 (44-68) years; median age at diagnosis was 61 (46-74) years. Three patients presented with progressively worsening shortness of breath and chest tightness on exertion, six suffered with gradually progressive neuropathy, one was hospitalised with heart failure and oedema whilst one was asymptomatic but had a family history of FAP. Amyloid deposits were identified in biopsies in all cases by Congo red staining and were confirmed to be of ATTR type by immunohistochemistry. Genetic analysis revealed five novel variants: p.E74L (E54L), p.E74Q (E54Q), p.A101V (A81V), p.H110D (H90D) and p.I127F (I107F). The cohort was followed for a median (range) of 4 (1-7) years after referral during which six patients died of cardiac amyloidosis. Median (range) age at death was 66 (60-80) years, and median (range) time from onset of symptoms to death was 8 (4-16) years.

In silico analyses showed small structural changes associated with each of the novel TTR variants. Thus we speculate that these molecular modifications may have contributed to their amyloidogenic properties. Furthermore, we identified the substitutions resulting in the p.E74L/Q (E54L/Q) to likely have a particularly deleterious impact on the TTR molecule. Multiple sequence alignment revealed the native p.E74 (E54) amino acid to be evolutionary highly conserved with no alternation of this residue among other species, indicating that it must be structurally and functionally important. Indeed, the side chain of the p.E74 (E54) forms hydrogen bonds with thyroxine, thus plays vital role in its transport. Replacement of this negatively charged, polar glutamic acid with the hydrophobic leucine residue may abolish this interaction.

## Conclusions

We describe the clinical phenotypes and disease course in 11 patients with novel TTR mutations. Each variant was found in at least two unrelated individuals who had similar clinical features, which make it highly unlikely to be an incidental finding. Immunohistochemistry confirmed that amyloid fibrils were composed of TTR, which in conjunction with the characteristic clinical features of ATTR amyloidosis manifesting with cardiomyopathy and neuropathy, indicates that these novel TTR mutations were indeed the cause of ATTR amyloidosis in our patients.

